# Acupuncture for Cancer-Induced Bone Pain?

**DOI:** 10.1093/ecam/neq020

**Published:** 2011-03-10

**Authors:** Carole A. Paley, Michael I. Bennett, Mark I. Johnson

**Affiliations:** ^1^Faculty of Health, Leeds Metropolitan University, Leeds, UK; ^2^Airedale General Hospital, Keighley, UK; ^3^Leeds Pallium Research Group, Leeds, UK; ^4^Lancaster University, International Observatory on End of Life Care, Lancaster, UK

## Abstract

Bone pain is the most common type of pain in cancer. Bony metastases are common in advanced cancers, particularly in multiple myeloma, breast, prostate or lung cancer. Current pain-relieving strategies include the use of opioid-based analgesia, bisphosphonates and radiotherapy. Although patients experience some pain relief, these interventions may produce unacceptable side-effects which inevitably affect the quality of life. Acupuncture may represent a potentially valuable adjunct to existing strategies for pain relief and it is known to be relatively free of harmful side-effects. Although acupuncture is used in palliative care settings for all types of cancer pain the evidence-base is sparse and inconclusive and there is very little evidence to show its effectiveness in relieving cancer-induced bone pain (CIBP). The aim of this critical review is to consider the known physiological effects of acupuncture and discuss these in the context of the pathophysiology of malignant bone pain. The aim of future research should be to produce an effective protocol for treating CIBP with acupuncture based on a sound, evidence-based rationale. The physiological mechanisms presented in this review suggest that this is a realistic objective.

## 1. Introduction

Bone pain is the most common type of pain in cancer. Bony metastases are common in advanced cancers, particularly in multiple myeloma, breast, prostate or lung cancer and they occur more commonly on the axial skeleton [1, 2]. Nowadays, the survival rate of many patients after diagnosis of bone metastases is relatively long, particularly in breast and prostate cancers where survival may be measured in years [1, 3]. Therefore it is important to control pain and preserve function to enable these patients to enjoy as high a quality of life as possible [4].

Current pain-relieving strategies include the use of opioid-based analgesia, bisphosphonates and radiotherapy. Although patients experience some pain relief, these interventions may produce unacceptable side-effects. In particular, the use of opioid-based analgesia commonly results in side-effects including constipation, nausea and drowsiness. A common symptom experienced by patients with cancer-induced bone pain (CIBP) is spontaneous breakthrough pain, which is severe and difficult to control because the levels of medication required for pain relief would be unacceptable [2, 5].

Acupuncture may represent a potentially valuable adjunct to existing strategies for pain relief and is known to be relatively free of harmful side-effects [6–8]; however, although acupuncture is widely used in palliative care settings [9] for all types of cancer pain the evidence-base is sparse and inconclusive [10]. Furthermore, safety guidelines for the use of acupuncture treatment in cancer patients exist [11], yet there have been no studies specifically looking at adverse events in patients with bony metastases and no high-quality randomized controlled trials have been conducted to investigate the effects of acupuncture on CIBP. Acupuncture is available widely in the palliative care setting [9] but the evidence-base is poor. The most recent systematic review of acupuncture for cancer pain was inconclusive and did not specifically mention CIBP [12]. We are in the process of undertaking a Cochrane Systematic Review of acupuncture for cancer pain, and our literature search, which has incorporated detailed search strategies of seven major databases and includes searches of integrative and complementary medicine journals, has found no randomized controlled trials specifically evaluating acupuncture as a treatment for CIBP [13]. Furthermore, our review of literature has not revealed any other types of studies in humans either investigating the effects of acupuncture on CIBP or providing a comprehensive scientific debate on its potential as a treatment for this type of pain. However, recent animal studies have investigated the effects of electroacupuncture on rat models of CIBP and these give us an insight into the mechanisms involved in mediating acupuncture-induced analgesia [14–16]. The aim of this critical review is to consider the known physiological effects of acupuncture and discuss these in the context of the pathophysiology of malignant bone pain.

## 2. Physiological Rationale for Using Acupuncture to Reduce CIBP

### 2.1. The Mechanisms Underlying CIBP

The physiological mechanisms of bone pain are not fully understood [17], but the pathophysiology of bony metastases suggests that the following factors are likely to be causative [5]:

- The release of chemical mediators, such as cytokines, from tumor cells.
- Increased pressure within the bone.
- Microfractures.
- Periosteal stretching.
- Muscle spasm.
- Nerve root infiltration.
- Nerve compression (due to vertebral collapse).

**Figure 1:**
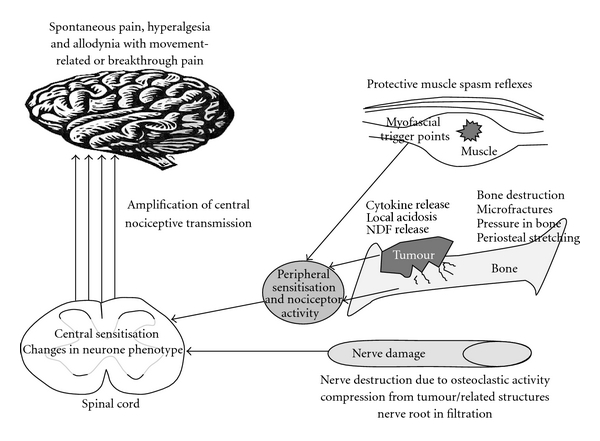
Mechanisms of cancer-induced bone pain.

It is likely that a combination of peripheral and central mechanisms contributes to pain associated with bony metastases and these are described below and illustrated in Figure 1.

#### 2.1.1. Peripheral Mechanisms

periosteum is well innervated and the sensory free nerve endings present are thought to be involved in the mediation of pain. Although the precise mechanisms are unclear, it is thought that excessive osteoclast-induced bone destruction leads to pain through stimulation of mechanoreceptors in the periosteum [18]. Sensory nerve fibers may also be destroyed due to excessive osteoclastic activity and this may lead to neuropathic-type pain [18]. Sensory neurons in the periosteum are also affected by increased mechanical stress within the bone which may lead to pain and increased sensitization [19]. This effect is enhanced by the presence of inflammatory cells within the tumor caused by an osteoblastic response, contributing to a local acidosis and in turn sensitizing acid-sensing ion channels such as the transient receptor potential vanilloid receptor 1 (TRPV1). Studies have shown that TRVP1 is important in the integrative signaling in CIBP and inhibiting its release is important in controlling complex pain states [20]. Tumor cells and tumor stromal cells secrete various inflammatory cells that contribute to the sensitization of excitation of primary afferent neurons, causing peripheral sensitization. One product that is significant in CIBP is nerve growth factor (NGF), which also acts to intensify nociceptive transmission in the dorsal horn [21].

#### 2.1.2. Central Mechanisms

It has been suggested that CIBP is a unique pain state showing physiological characteristics of both inflammatory and neuropathic pain and changes in dorsal horn cell phenotype [21–24]. Whereas inflammatory pain states are characterized by up-regulation of calcitonin gene-related peptide (CGRP), and neuropathic pain states by up-regulation of neuropeptide Y (NPY), CIBP is distinct in that there is an up-regulation of dynorphin in the deep dorsal horn and increased expression of c-fos [23, 25]. In addition, it has been shown in rat models of CIBP that repetitive noxious stimulation of primary C-fiber afferents results in a “wind-up” phenomenon which is characterized by an escalation of nociceptive transmission by cells in the dorsal horn [23, 24]. Wind-up contributes to central sensitization via wide-dynamic range (WDR) cells resulting in the amplification and prolongation of nociceptive transmission in ascending pathways in the central nervous system. This sensitizing effect results in sensitivity to normally non-noxious mechanical and thermal stimuli (allodynia) and exaggerated responses to noxious stimuli (hyperalgesia). Central sensitization is mediated via activation of the *N*-methyl-d-aspartate (NMDA) glutamate receptor and is a feature of many chronic pain states.

Studies using rat models of CIBP have also shown that the proportion of WDR neurons almost doubles in the presence of bony metastases. The proportion of nociceptive-specific (NS) to WDR neurons in normal controls is 74 : 26%, and changes to 53 : 47%, respectively, during CIBP [23]. This means that neurons which normally respond to noxious stimuli start to respond to non-noxious stimuli resulting in allodynia and hyperalgesia [23, 26]. Thus, central sensitization and changes in phenotype of dorsal horn neurons will result in hypersensitivity to mechanical input and be responsible for movement-related pain which is characteristic of CIBP.

It has been shown that CIBP causes a decrease in *μ*-opioid receptors in dorsal root ganglia and this may explain the decreased response to opiate drugs such as morphine. This response was not shown in inflammatory models, where morphine was effective in reducing pain [17]. CIBP-induced changes in the endogenous opioid system may also affect structures on the descending pain inhibitory pathways which are involved in regulating spinal responses to pain via sensory input [27], for example, the periaqueductal gray (PAG) and parabrachial areas of the brain that release dynorphin and in turn activate the serotogenic system within the brainstem and spinal cord.

The multifactorial mechanisms underlying CIBP probably explain why the level of pain experienced by patients does not necessarily correspond with the extent of the bony metastases [18, 19]. Coupled with affective and cognitive factors associated with the prognosis of CIBP, this presents a complex pain state requiring a multidisciplinary approach to pain management and a unique problem when considering appropriate mechanisms of analgesia.

### 2.2. How Can Acupuncture Influence the Physiology of CIBP?

**Figure 2:**
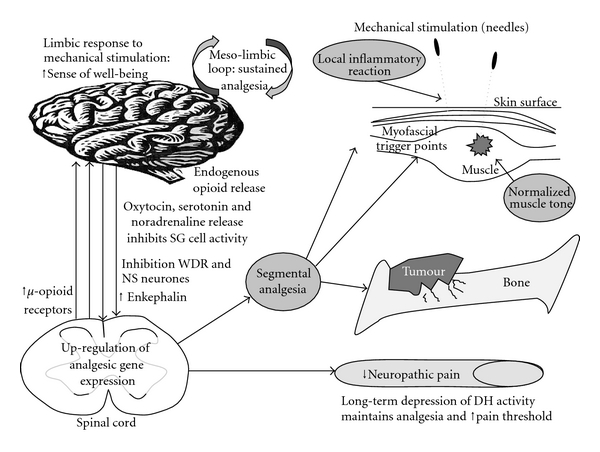
Mechanisms of acupuncture analgesia.

Many of the studies investigating the physiological effects of acupuncture have been conducted in the laboratory and a significant number have used animal subjects [14–16, 28–30]. Because so little clinical evidence exists we have had to rely on these laboratory studies for our discussion, yet they have shortcomings in relation to clinical practice because many “human” factors play a part in the therapeutic process and the ideal conditions of the laboratory cannot be reproduced in the clinic. However, these studies provide us with useful guidance on which mechanisms may act to produce the desired therapeutic effect, and the clinician must use skill and judgment to adapt these guidelines according to the individual patient. Acupuncture is likely to be effective in treating CIBP if it can reduce pain transmission and/or sensitization at peripheral and central sites (Figure 2).

#### 2.2.1. Segmental Analgesia and Desensitization

Acupuncture stimulates small myelinated A*δ* fibers in muscle and skin which synapse with interneurones in the substantia gelatinosa (SG) of the spinal cord resulting in the release of inhibitory neuromodulators such as enkephalin that reduce activity in NS and WDR neurons [31]. It is claimed that it is necessary to retain the needles *in situ* for 10–20 min to ensure the release of enkephalin [32]. Reduced nociceptive transmission in the dorsal horn following A*δ* input (long-term depression) has been shown to outlast the stimulation by hours [33, 34].

Continuous or repetitious stimulation of A*δ* fibers using electrical currents has been shown to reduce sensitization of postsynaptic receptors and/or a decrease in neurotransmitter release in the dorsal horn in normal rats [34]. Other studies have used rat models of CIBP to investigate the effects of electroacupuncture on cancer-induced hyperalgesia [14, 15]. Daily treatment for 30 min at 10 Hz/2 mA/0.4 ms pulse for 5 days suppressed expression of dynorphin in the dorsal horn of rats with artificially induced bone cancer [15]. Dynorphin is known to facilitate hyperalgesia and it was suggested that suppression of this may inhibit CIBP, as was demonstrated in the rat model. In a similar study, interleukin-1**β** expression was measured in rats with CIBP-related hyperalgesia, as this is related to the maintenance of persistent pain [14]. The data suggested that because electroacupuncture suppressed interleukin-1**β** expression it would therefore be an effective treatment for the treatment of CIBP. Another recent animal study used a neuropathic cancer pain model in mice and found that electroacupuncture decreased levels of substance P in the dorsal horn but increased levels of *β*-endorphin in the blood by 51.5% and in the brain by 12.6%, suggesting that it may be useful as an alternative treatment for this type of cancer-related pain [16]. In all the animal studies, however, it must be borne in mind that the doses of electroacupuncture applied in the laboratory represent “ideal” conditions which might not be possible to attain in the clinic, and the findings of these studies have yet to be applied to human subjects.

Further animal studies have shown that acupuncture [33–35], electroacupuncture [33–35] and transcutaneous electrical nerve stimulation (TENS) [34, 36] reduce central sensitization and may therefore be useful in CIBP [37]. Regular treatment at a low-frequency which maintains analgesia and produces a long-term depression in the dorsal horn may be achieved by stimulation of afferent A*δ* fibers using manual acupuncture alone and studies using rats have shown that this depression may last for days or weeks [33, 35]. Recent studies using mice have shown that manual acupuncture with bidirectional stimulation stimulates connective tissue which generates a strong afferent input to the dorsal horn and is therefore likely produce a depressing effect [28, 38]. However, these connective tissue responses have yet to be reproduced in the human population to determine whether they are related to therapeutic effects. It has also been suggested that the release of oxytocin in response to non-noxious sensory stimulation such as manual acupuncture may give rise to long-term elevations in pain threshold [35]. It is therefore likely that acupuncture should help to prevent wind-up, central sensitization and the resultant allodynia and hyperalgesia.

The need for regular and repetitive stimulation of afferent A*δ* fibers to achieve long-lasting analgesia is borne out by anecdotal evidence, which suggests that patients with cancer pain need to be treated two or three times per week initially for optimal effects, particularly in cases of CIBP where pain relief is often achieved for a very short time [39]. For treating CIBP, consideration should be given to the segmental innervations (sclerotomes) of bones affected by metastatic deposits. Application of acupuncture needles to the muscles corresponding with the same spinal segment (myotomes) should result in analgesia throughout the segment. Also, as each afferent nerve originates from more than one spinal segment, the application of needles from adjacent segments will increase the effect of the stimulation.

#### 2.2.2. Extrasegmental Effects

The cumulative response of acupuncture may also be demonstrated by permanent changes in the opioid peptide mechanism which enhances gene expression. The arcuate nucleus of the hypothalamus is activated by peripheral A*δ* fiber input resulting from acupuncture and *β*-endorphin is released, which has an effect on the PAG via the descending pain inhibitory system. This is similar to the effect of opioid drugs in stimulating the release of serotonin and met-enkephalin that inhibit the SG cells, and is supported by clinical trials which showed that naxolone reverses some of the pain-relieving effects of acupuncture and electroacupuncture [30, 40–42].

The relatively sustained effects of acupuncture analgesia might be due to an up-regulation in analgesic gene expression, which demonstrates the importance of regular “top-ups”. It has also been suggested that sustained analgesia may be maintained by a positive feedback loop in the meso-limbic system which results in a continuous outflow from descending inhibitory pathways [31, 43]. Cumulative stimulation of A*δ* fibers also results in increased amounts of peptides being manufactured and stored, therefore prolonging the analgesic response to acupuncture. Furthermore, up-regulation of the opioid peptide mechanism as a cumulative result of A*δ* stimulation has been found to activate *μ*-opioid receptors and increase their binding potential, thus overriding central changes to the endogenous opioid system in CIBP [44].

Non-opioid systems also play an important part in acupuncture analgesia. The release of serotonin in the brainstem activates descending inhibitory systems resulting in the release of more serotonin and noradrenaline in the dorsal horn. Noradrenaline modulates pain transmission within the dorsal horn by inhibiting the post-synaptic membrane of SG cells, thus reinforcing the effects of the opioid-peptide mechanism [45].

An immediate and powerful sympathetic response occurs within a segmental distribution when needles are inserted. In the long-term, in addition to depressing pain transmission within the dorsal horn it reduces pain perception and produces an autonomic blockade by interrupting autonomic reflexes [32]. This is a cumulative effect occurring over several treatments. Acupuncture also affects autonomic activity in the hypothalamus and this might explain why patients feel relaxed and occasionally drowsy after treatment. The use of auricular (ear) acupuncture to elicit autonomic effects may also be useful for pain relief in CIBP but the precise mechanisms of this type of acupuncture remain unclear [36]. It is based on the assumption that all internal organs are represented on the ear, but unfortunately so many different maps exist that there is little agreement as to point location [46, 47]. There is some evidence that auricular acupuncture is useful for postoperative pain [47], cancer pain [48] and for vasomotor symptoms associated with hormone therapy in cancer patients [49], but not CIBP and therefore this would be an interesting area for further research.

#### 2.2.3. Central Effects

Functional brain imaging studies have demonstrated that the application of acupuncture affects the limbic system, which modulates emotional (affective) responses to pain [50–54]. The way acupuncture affects the emotional component of CIBP has emerged as a result of brain imaging studies, which show that sensory input may be modulated by the emotional reaction and cognitive aspects of pain and vice versa [55–57]. This is borne out by recent studies investigating the mechanisms of placebo analgesia [58–60].

The effect of acupuncture on the limbic system might also explain why some patients (strong reactors) experience a euphoric reaction to acupuncture. The phenotypic changes in lamina I neurons in the dorsal horn mentioned earlier are also likely to contribute to the affective and autonomic responses associated with CIBP [23]. The central modulation of pain is important in CIBP because it reduces the unpleasantness of pain and affects how well patients tolerate it. One of the key features of this is the C fiber tactile system where light stimulation of the skin stimulates mechanoreceptors and induces a response in the limbic system. This reduces the affective component of pain and increases the sense of well-being [61], and for this reason it may be that point specificity is not as important for central pain modulation as it is in segmental acupuncture [62].

#### 2.2.4. Myofascial Trigger Points

Another manifestation of chronic pain is the presence of reactive muscle spasm which in turn causes additional pain and shortening of the muscle, affecting function [63]. The use of acupuncture for myofascial trigger points (MTPs) may be a useful way of both alleviating discomfort and improving functional status. A recent study using anesthetic injected into MTPs in chronic neck pain has confirmed that function can be improved by eradicating these points [64]. It was suggested that MTPs serve to perpetuate lowered pain thresholds in uninjured tissues and that central sensitization may itself be perpetuated by the presence of MTPs [64]. Where MTPs are present as a result of CIBP they are likely to perpetuate themselves by the continual release of acetylcholine (ACh) causing a sustained contraction, which increases energy demands in the muscle but impedes the circulation. Nociceptive neurotransmitters are released and it is thought that this has a sensitizing effect on the muscle endplates which in turn contribute to the continued release of ACh in a feedback loop [32]. The use of acupuncture to normalize muscle tone may break this feedback loop and reduce perpetual central sensitization.

The effect of acupuncture on MTPs in normalizing muscle tone, restoring muscle balance and thus improving mobility and function has been noted in cancer patients and is therefore an important outcome for patients suffering from CIBP [65].

### 2.3. The Clinical Use of Acupuncture in CIBP

#### 2.3.1. Prevalence of Acupuncture for CIBP

There is evidence to suggest that acupuncture is widely used in palliative cancer care [9, 39, 66, 67], and large surveys show that a relatively high percentage of cancer patients use various complementary therapies for pain and other symptoms [68–70]. Clinical experience suggests that acupuncture is effective for treating CIBP, although there is no published information indicating the extent of its use in practice.

#### 2.3.2. Safety of Acupuncture in CIBP

Although studies have shown acupuncture to be relatively safe from serious adverse effects [7, 8] the unique characteristics of CIBP require careful consideration. The presence of hyperalgesia or allodynia as a result of CIBP presents potential problems for the acupuncturist in that patients might not tolerate the treatment. Any short-term exacerbation of pain as a result of acupuncture may not be tolerable for patients who are already overburdened with severe pain and therefore caution is required with patients who are likely to be strong reactors [32, 71]. It is suggested that strong reactors are often individuals who are more influenced by sensory stimulation and may be artistic or inclined toward religious belief, although there is little published evidence of this [72, 73]. As with all new treatments, acupuncture should be introduced cautiously at first so the reactions of the patient may be monitored. It must be said, however, that strong reactors are often good responders, even with minimal stimulation, and therefore it might only be necessary to insert the needles for a very short time to achieve the desired amount of stimulation [74].

Patients undergoing cancer treatment such as chemotherapy can become severely immunosuppressed and potentially more vulnerable to infection and bleeding due to low platelet counts. This represents a known risk but is regarded as an unlikely occurrence provided safety guidelines are followed and practitioners liaise closely with oncology staff and maintain a close scrutiny of blood counts [11, 71].

The insertion of needles causes tissue damage and the resulting inflammation increases local blood flow which is normally a desired effect because it promotes tissue healing. However, in cancer an increase in blood flow might not be desirable, particularly around the site of a tumor because it could encourage proliferation or metastatic spread (although we have found no evidence for this). Caution must also be used when lymphedema is present due to a risk of infection and cellulitis [71] and it is recommended that the contralateral limb is needled in these cases [11]. If acupuncture is effective in alleviating pain, one might assume that it could mask tumor progression. However, based on clinical experience it has been suggested that where patients who have been previously responsive to acupuncture suddenly stop responding, this might be a sign that there is metastatic spread and they should therefore be checked for disease progression [11, 32, 71, 75].

The effect of acupuncture on MTPs and muscle spasm must be borne in mind when needling around the spine. Muscle spasm may be acting to protect spinal instability due to metastatic growth and the reduction of this spasm by acupuncture may result in spinal collapse and cord transaction, though we have found no published evidence of this [11, 71]. Although evidence suggests that the direct risk of adverse events due to acupuncture in patients with CIBP is relatively low, practitioners must keep in mind the “indirect” risk that patients may regard acupuncture as a true alternative to conventional treatments and suffer unduly as a result [70].

#### 2.3.3. Clinical Efficacy and Effectiveness

The extent of the use of acupuncture in CIBP is not known although anecdotal evidence and preliminary clinical data are available. In a small, uncontrolled trial (*n* = 28) of four groups of palliative care patients, including patients with neuropathic pain, benign soft tissue pain, benign bone pain and malignant bone pain, significant pain relief was achieved in all groups using auricular stud acupuncture [67]. Clinical data collected from a UK hospice over 12 months showed that 10 out of 15 patients with bone pain experienced either “excellent” or “good” pain relief following treatment with acupuncture, although no details of treatment protocols were given [66]. Filshie [39] collected clinical data over a period of 5 years involving 156 patients who attended cancer clinics and were treated with acupuncture. The results of the treatment were promising, although it was stated that acupuncture is only useful in “selected patients” with bone pain. No further details were given on which patients with bone pain might benefit from acupuncture.

The most recent systematic review on acupuncture for cancer-related pain was inconclusive and none of the included studies used CIBP populations [12]. Recently, we have published a protocol for undertaking a Cochrane review on acupuncture for cancer pain in adults with particular emphasis on CIBP [13]. Our literature searches have revealed no high-quality evidence for the efficacy of acupuncture in treating CIBP in humans.

## 3. Summary: Future Research

CIBP is a unique pain state and is characterized by central sensitization and an “up-regulated” nociceptive system. Acupuncture is known to reduce central sensitization in the dorsal horn and to reduce transmission of nociceptive information. It is likely that regular treatments reduce ongoing nociceptive transmission and sensitization and up-regulate the opioid peptide system. The release of MTPs may also help to lower central sensation. Additionally, the effect of acupuncture on the limbic system in the brain may modulate emotional responses to pain and render it more tolerable. Future research must focus upon well-designed randomized controlled clinical trials using human subjects to specifically investigate the effect of acupuncture on CIBP and focus in particular upon aspects of needle placement and dosage for optimal results. The aim of such research should be to produce an effective protocol for treating CIBP based on a sound, evidence-based rationale. The physiological mechanisms presented here suggest that this is a realistic objective.
